# Schwarzinicine A inhibits transient receptor potential canonical channels and exhibits overt vasorelaxation effects

**DOI:** 10.1002/ptr.7489

**Published:** 2022-05-10

**Authors:** Yin‐Ying Mak, Bi‐Juin Loong, Paul Millns, Claudia C. Bauer, Robin S. Bon, Yvonne Mbaki, Fong‐Kai Lee, Kuan‐Hon Lim, Cin Kong, Sue‐Mian Then, Kang‐Nee Ting

**Affiliations:** ^1^ School of Pharmacy University of Nottingham Malaysia Semenyih Malaysia; ^2^ School of Life Sciences University of Nottingham Nottingham UK; ^3^ Department of Discovery and Translational Science, Leeds Institute of Cardiovascular and Metabolic Medicine University of Leeds Leeds UK

**Keywords:** calcium channel blocker, *Ficus schwarzii*, L‐type voltage‐gated calcium channels, schwarzinicine A, TRPC channels, vasorelaxation

## Abstract

This study investigated the vasorelaxant effects of schwarzinicine A, an alkaloid recently reported from *Ficus schwarzii* Koord. Regulation of calcium homeostasis in vascular smooth muscle cells (VSMC) is viewed as one of the main mechanisms for controlling blood pressure. L‐type voltage‐gated calcium channel (VGCC) blockers are commonly used for controlling hypertension. Recently, the transient receptor potential canonical (TRPC) channels were found in blood vessels of different animal species with evidence of their roles in the regulation of vascular contractility. In this study, we studied the mechanism of actions of schwarzinicine A focusing on its regulation of L‐type VGCC and TRPC channels. Schwarzinicine A exhibited the highest vasorelaxant effect (123.1%) compared to other calcium channel blockers. It also overtly attenuated calcium‐induced contractions of the rat isolated aortae in a calcium‐free environment showing its mechanism to inhibit calcium influx. Fluorometric intracellular calcium recordings confirmed its inhibition of hTRPC3‐, hTRPC4‐, hTRPC5‐ and hTRPC6‐mediated calcium influx into HEK cells with IC_50_ values of 3, 17, 19 and 7 μM, respectively. The evidence gathered in this study suggests that schwarzinicine A blocks multiple TRPC channels and L‐type VGCC to exert a significant vascular relaxation response.

**Abbreviations**
[Ca^2+^]_i_
: intracellular calcium
AM: acetoxymethyl ester
CaCl_2_
: calcium chloride
CO_2_
: carbon dioxide
CRC: concentration response curve
DAG: diacylglycerol
DRG: dorsal root ganglion
eNOS: endothelial nitric oxide synthase
GDNF: glial cell line‐derived neurotrophic factor
KCl: potassium chloride
L‐NAME: *N* ^*G*^‐Nitro‐L‐arginine methyl ester
MgSO_4_
: magnesium sulphate
NO: nitric oxide
O_2_
: oxygen
OAG: 1‐oleoyl‐2‐acetyl‐*sn*‐glycerol
PLC: phospholipase C
S1P: sphingosine‐1‐phosphate
SAG: 1‐stearoyl‐2‐arachidonyl‐*sn*‐glycerol
SBS: standard bath solution
SD: Sprague–Dawley
TRPC: transient receptor potential canonical
VGCC: voltage‐gated calcium channel
VSMC: vascular. Smooth muscle cell
WT: wild type

## 1 INTRODUCTION

**FIGURE 1:**
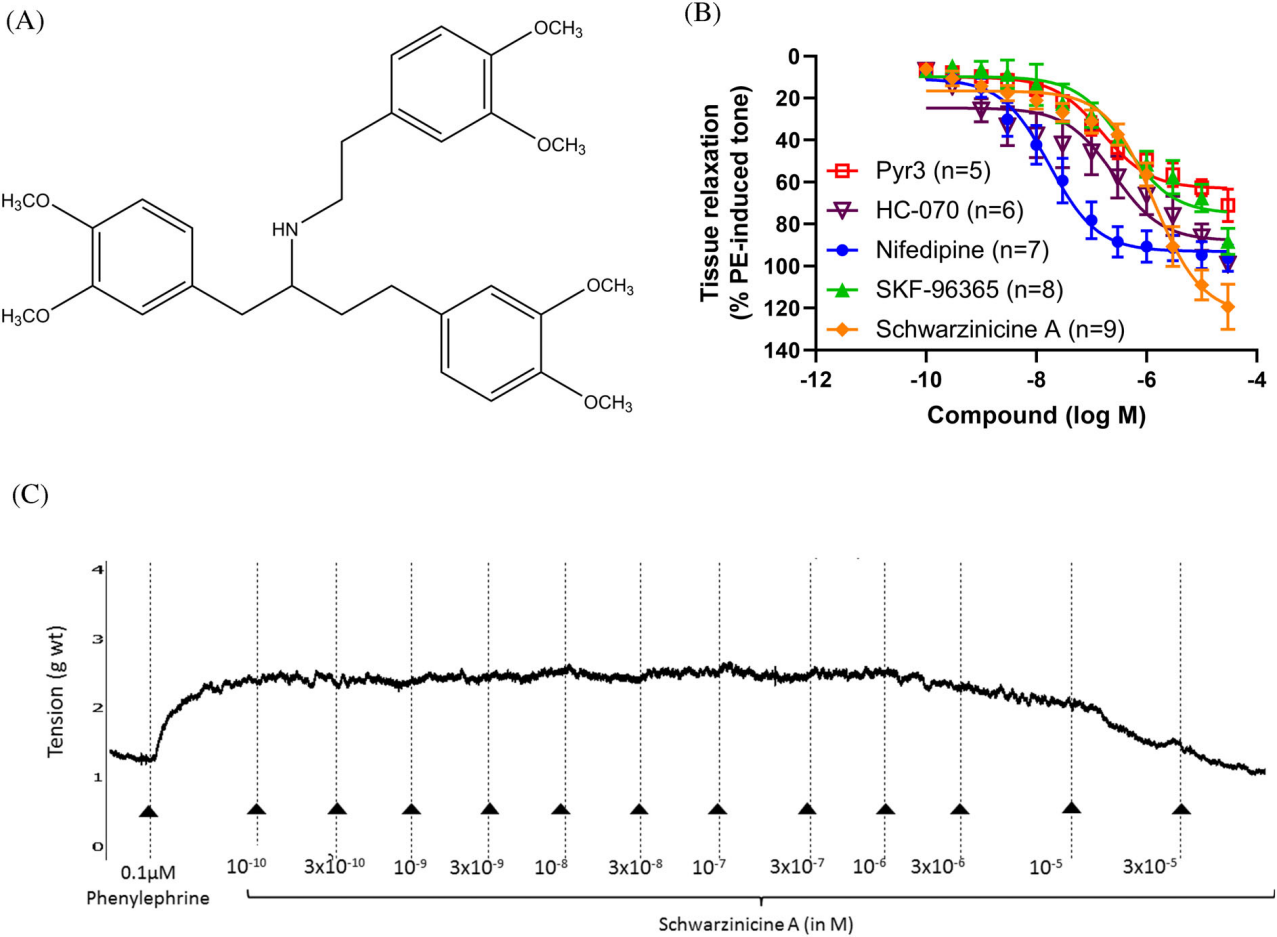
(a) Chemical structure of schwarzinicine A, which was obtained naturally as a scalemic mixture (4:1 ratio of (+)/(−)). (b) Cumulative concentration‐response curve of schwarzinicine A compared to vehicle control (DMSO), Pyr3, HC‐070, nifedipine and SKF96365. Tissue relaxations were expressed as the percentage of phenylephrine‐induced contraction. The data represent the mean values ± SEM of *n* number of animals. (c) Representative trace recording of schwarzinicine A in rat isolated aorta

Based on our ongoing search for biologically active natural products, we discovered schwarzinicine A (Figure 1a), which is a new 1,4‐diarylbutanoid–phenethylamine alkaloid, from the leaf extract of *Ficus schwarzii* Koord (Krishnan et al., 2020). Schwarzinicine A was found to exhibit marked vasorelaxation properties with a profile similar to dobutamine, a β‐adrenergic agonist which has similar phenylethylamine structure as schwarzinicine A (Krishnan et al., 2020). Following that, we reported a concise synthesis of schwarzinicine A, thus enabling further biological evaluation to be carried out (Lee et al., 2021).

The mainstay for treatment of hypertension is the use of L‐type voltage‐gated calcium channel (VGCC) blockers. Regulation of calcium homeostasis in vascular smooth muscle cells (VSMC) is viewed as one of the main mechanisms for controlling blood pressure. An important discovery of the transient receptor potential canonical (TRPC) family of proteins is that they were found to be present and functional in smooth muscle cells and endothelial cells of different animal species (Earley & Brayden, 2015).

Growing literature demonstrated that the molecular mechanisms of TRPC channels in modulating Ca^2+^ dynamics are complex in nature. For VSMCs contraction, a number of studies have suggested that TRPC‐mediated Ca^2+^ influx is through mechanisms involving direct Ca^2+^ transport via the pore region of these channels. This mechanism is proposed to be via interaction with L‐type VGCCs by altering the membrane potential or recruiting signalling messengers (Earley & Brayden, 2015; Kiselyov et al., 1998). Our research question was focusing on the direct inhibition of Ca^2+^ entry during TRPC channels activation which leads to vasorelaxation. On the other hand, the expression of endothelial TRPC channels promotes vasorelaxation through a different pathway – association with BK_Ca_ channel which induces membrane hyperpolarisation in VSMCs (Earley & Brayden, 2015; Kochukov, Balasubramanian, Noel, & Marrelli, 2012; Kwan et al., 2009; Martín‐Bórnez, Galeano‐Otero, Del Toro, & Smani, 2020). It is suggested that these downstream relaxation effects following Ca^2+^ influx prevent prolonged myogenic contraction.

The importance of these channels in the cardiovascular system has been clearly demonstrated in the last decade (Earley & Brayden, 2015; Wang et al., 2020). Changes to these TRPC channels have been linked to vascular pathologies such as hypertension and pulmonary oedema. The TRPC channels are a group of receptor‐operated calcium‐permeable non‐selective cation channels of the TRP superfamily. They are activated by the phospholipase C (PLC) pathway (Wang et al., 2020) and inhibited by SKF96365 (Harteneck, Klose, & Krautwurst, 2011). Current evidence indicates that TRPC1, TRPC3, TRPC4 and TRPC6 channels are functionally important in regulating vascular contractility via the regulation of calcium dynamics (Kunichika et al., 2004; Liu, Xiong, & Zhu, 2014). TRPC1, 3, 4, 5 and 6 protein expressions were detected in various vascular beds, including cerebral and coronary arteries (Yip et al., 2004), Sprague–Dawley (SD) rat resistance arteries (Welsh, Morielli, Nelson, & Brayden, 2002), thoracic aorta (Facemire, Mohler, & Arendshorst, 2004) and embryonic rat thoracic aortic smooth muscle cells (A7r5 cells) (Jung, Strotmann, Schultz, & Plant, 2002). Interestingly, the expression of TRPC channels was found to increase in VSMCs isolated from hypertensive animals and patients (Chen et al., 2010; Dietrich et al., 2005). The development of the functional characterisation and pharmacology of these TRPC channels has been slow until recently, when a number of novel selective small molecules have been discovered (Wang et al., 2020).

Following these initial observations, the present study was designed to further investigate schwarzinicine A's potential role as a calcium channel modulator and to characterise its pharmacological effects using the rat isolated aorta as a native system. We hypothesised that schwarzinicine A modulates calcium dynamics across the cell membrane with the involvement of TRPC channels and possibly L‐type VGCCs.

## 2 METHODS

### 2.1 Isometric tension recordings

#### 2.1.1 Tissue preparation

Aorta isolated from adult male SD rats (240–480 g, 8–10 weeks old) were used in isometric tension recordings. SD rat was used as a model as it is an outbred stock which abundantly expresses TRPC 3, 4, 5 and 6 mRNA and protein in its aorta (Facemire et al., 2004). The aorta was excised in 2 mm ring and transferred into fresh Krebs‐Ringer bicarbonate solution. The organ bath containing fresh isolated aortic tissues was assembled and tested for tissue viability as described in Loong, Tan, Lim, Mbaki, and Ting (2015).

#### 2.1.2 Effects of schwarzinicine A and other calcium channel inhibitors against rat isolated aortae

Endothelium‐intact aorta was pre‐constricted with 0.1 μM phenylephrine. The concentration of phenylephrine used elicited at least 70% submaximal of phenylephrine‐induced tissue contraction. Once a stable tone was established, cumulative concentration response curves (CRCs) of concentration range from 0.1 nM to 30 μM were generated for schwarzinicine A. Each concentration was added every 7 min or when the tissue response had plateaued.

The experiment was repeated using HC‐070 (a selective, nanomolar TRPC4/5 channel inhibitor with micromolar activity against TRPC3), SKF96365 (TRPC channel blocker), Pyr3 (TRPC3‐selective channel blocker), nifedipine (L‐type calcium channel blocker) and DMSO (vehicle control).

#### 2.1.3 Effects of schwarzinicine A on calcium chloride (CaCl_2_ )‐induced contraction in calcium‐free Krebs solution

Endothelium‐intact aorta was tested for tissue viability with 60 mM KCl twice in calcium‐containing Krebs solution (Loong et al., 2015). Then, the aorta was equilibrated in calcium‐free Krebs solution for 30 min before incubating the tissues further with different concentrations of schwarzinicine A (3, 10, 30 μM) for another 30 min. These three concentrations were chosen based on the earlier generated CRC (see Figure 1b). Following a single application of 60 mM KCl to depolarise VGCC in calcium‐free Krebs solution, a CRC to CaCl_2_ (1 μM to 3 mM) was carried out.

### 2.2 Calcium imaging of DRG neurons

Two SD rats (250–300 g) were euthanised by CO_2_ exposure and then cervical dislocation. Isolation of DRG neurones, preparation of coverslips and loading of 5 μM fura 2‐ acetoxymethyl ester (AM) were performed as described previously (Lee et al., 2018).

In a field of 30–40 neuronal cells, the [Ca^2+^]_i_ of individual neurones were estimated as the change in the ratios of peak fluorescence intensities (measured at 500 nm) at excitation wavelengths of 340 and 380 nm, respectively (Andor iQ imaging software, Hamamatsu Orca II camera, Leica DMIRB microscope). The mean diameter of the cells (*n* = 535) was 27.5 ± 0.2 mm. DRG neurones were superfused (2 ml/min) with 30 mM KCl (in superfusion buffer, 60 s) to evoke a depolarisation‐induced calcium influx. This was represented as the control response. It was followed by a 45‐min washout period, before the direct addition of 1 ml schwarzinicine A (30 μM) for 4 min and later addition of combined 30 mM KCl and 30 μM schwarzinicine A. Similar experiments were repeated by replacing schwarzinicine A with 30 μM nifedipine.

### 2.3 Intracellular calcium measurements

#### 2.3.1 Cell culture

To study the inhibitory role of schwarzinicine A against TRPC channels, HEK cells overexpressing human TRPC channels (TRPC3, TRPC4, TRPC5 or TRPC6) were used. These channel subtypes were chosen based on their functional roles in vascular regulation reported in a number of studies (Álvarez‐Miguel, Cidad, Pérez‐García, & López‐López, 2017; Kochukov et al., 2012).

The methods used in cell culture, transfection and fluorometric intracellular calcium recordings were adopted from Minard et al. (2019). Wild‐type (WT) HEK 293 cells and (Tet+) HEK T‐Rex™ cells expressing either human TRPC3, TRPC4 or TRPC5 were cultured in 75 cm^2^ flasks in DMEM GlutaMAX™ (Invitrogen, Paisley, UK), supplemented with 10% FBS (Sigma‐Aldrich) and 100 units/ml, 100 μg/ml penicillin–streptomycin (ThermoFisher Scientific, UK). (Tet+) HEK T‐REx™ cells expressing TRPC channel proteins were supplemented with additional blasticidin (10 μg/ml) and zeocin (400 μg/ml; Invivogen, San Diego, California, USA) to maintain a stable expression of the tetracycline repressor protein and relevant TRPC channel. WT HEK 293 cells (ATCC) used for all cell culture experiments were from passage 23 to 35. HEK T‐REx™ cells (ThermoFisher Scientific) TRPC3 cells were cultured from passage 11 to 23, TRPC4 cells from passage 12–16, and TRPC5 cells from passage 36–40.

#### 2.3.2 Transfection (hTRPC6)

1 × 10^6^ WT HEK 293 cells suspended in 2 ml of media per well (≈ 40–60% confluence) were plated in a poly‐D‐lysine‐coated 6‐well culture dish (Corning Incorporated, USA) 24 hours before transfection. The cells were grown in a humidified incubator with 5% CO_2_ at 37°C overnight. The next day, cells were transfected using human TRPC6 cloned into pcDNA3 (stock 1.15 μg/μl) and jetPRIME® transfection reagent (VWR, Lutterworth, UK) in a ratio of 1:3. TRPC6 cloned into pcDNA3 was obtained from Dr Melanie Ludlow (Beech Lab, Leeds Institute of Cardiovascular and Metabolic Medicine, University of Leeds). Transfection mix was prepared by diluting 2 μg of hTRPC6 in 200 μl jetPRIME® buffer and 6 μl of jetPRIME® transfection reagent. After a brief vortex, the mix was incubated at room temperature for 10 min before being added to the cells. The cells were incubated for 24 hours before being plated.

#### 2.3.3 Fluorometric intracellular calcium recordings

Twenty four hours before the experiment, all cells were plated at 50,000 cells per well (≈ 90% confluence), onto black, clear‐bottom poly‐D‐lysine coated 96‐well plate. For hTRPC3/4/5‐expressing (Tet+) HEK T‐REx™ cells tetracycline (1 μg/ml, 1:1000 dilution) was added to the cells to induce the overexpression of the relevant TRPC protein. In the darkroom, media was removed before loading 50 μl of standard bath solution (SBS), which contained 2 μM fura 2‐AM and 0.01% pluronic acid to the cells. The cells were incubated for 1 hour at 37°C. After that, fura‐2 a.m. was removed and the cells were washed twice with 100 μl of fresh SBS. This step was followed by the addition of 80 μl recording buffer. Recording buffer consisted of SBS containing schwarzinicine A (0.1–100 μM) or vehicle (DMSO 0.1%). The cells were then incubated at room temperature for another 30 min before being assayed.

[Ca^2+^]_i_ recordings were carried out on FlexStation 3 Multimode Microplate Reader (Molecular Devices, San Jose, CA). Dual excitation wavelength (340 and 380 nM) and an emission wavelength of 510 nM were used in the experiment. Measurements were taken every five seconds for five minutes at room temperature. Baseline recordings were measured at the beginning from 0–55 s. At 60 s, 80 μl of compound buffer containing 200 μM OAG (TRPC3/6 channel activator) or 10 μM sphingosine‐1‐phosphate (S1P; TRPC4/5 activator) was automatically pipetted from the compound plate to the cell plate. Schwarzinicine A or DMSO 0.1% were previously added to the compound buffer preparation to maintain concentration on cells after compound addition. For TRPC3 and TRPC6 experiments, 0.01% pluronic acid was added to recording and compound buffers. Each fluorometric calcium recording experiment consisted of six technical replicates (six wells on each row of a 96‐well plate), represented by *N* = 6.

### 2.4 Materials and chemicals

Schwarzinicine A (*N*‐(3,4‐dimethoxyphenethyl)‐1,4‐bis(3,4‐dimethoxyphenyl)butan‐2‐amine) was previously obtained from the leaf extract of *Ficus schwarzii* (Krishnan et al., 2020) and was determined to be analytically pure based on ^1^H and ^13^C NMR analysis (purity ≥99%, by HPLC analysis). Lower concentrations of schwarzinicine A (0.1 nM to 30 mM; MW: 509.64 g/mol) were diluted accordingly from 100 mM stock solutions using 100% DMSO. All chemicals were dissolved in 100% DMSO unless stated otherwise. Fura‐2 a.m. (ThermoFisher Scientific, UK) were prepared as 1 mM stock solution. Nifedipine (Nacalai Tesque, Japan) was made into stock concentration of 10 mM. HC‐070 (Univeristy of Leeds, UK), OAG (Sigma‐Aldrich, UK), Pyr3 (Sigma‐Aldrich, US), and SKF96365 (Sigma‐Aldrich, US) were dissolved to make 100 mM stock solution. S1P (Sigma‐Aldrich, UK) was dissolved in methanol to 5 mM and stored at −80°C. Stock concentration of 10 mM (*R*)‐(−)‐phenylephrine hydrochloride (Sigma‐Aldrich, US) and 3 M KCl were dissolved in distilled water. Schwarzinicine A and other DMSO‐dissolved drugs were diluted ten10fold with descending DMSO content from 50% (10 mM), 25% (1 mM) to purified water (100 μM and below). The final concentration of DMSO did not exceed 0.15%, while methanol did not exceed 0.1%.

The Krebs‐Ringer bicarbonate solution was freshly prepared daily following the composition (in mM): NaCl 120, KCl 5.4, MgSO_4_ 1.2, KH_2_PO_4_ 1.2, NaHCO_3_ 25, D‐glucose 11.7, CaCl_2_ 1.26 and gassed with 95% O_2_, 5% CO_2_. Calcium‐free Krebs solution was prepared with CaCl_2_ omitted. The superfusion buffer used in calcium imaging was prepared following the composition (in mM): NaCl 145; KCl 5; MgSO_4_ 1; glucose 10; CaCl_2_ 2; HEPES 10 at pH 7.4. In fluorometric [Ca^2+^]_i_ recordings, SBS was prepared following the composition (in mM): NaCl 130, KCl 5, MgCl_2_ 1.2, glucose 8, CaCl_2_ 1.5 and HEPES 10 at pH 7.4.

### 2.5 The animal welfare and ethical statement

Ethics approval was obtained from the University of Nottingham's Animal Welfare and Ethics Review Body (AWERB) (Reference: UNMC#2kn). The rats were purchased from the animal house in University Putra Malaysia, Malaysia. They were given adequate food and water and were kept between 18 to 20°C in a 12‐hour light–dark cycle. The rats were anaesthetised with diethyl ether and sacrificed by cervical dislocation according to the methods described in Schedule 1 of the UK Animals (Scientific Procedures) Act 1986. Experimental procedures for DRG calcium imaging were carried out under Home Office Licences 40/3647 in accordance with the Animals (Scientific Procedures) Act 1986 and IASP guidelines (PPL No. 40/3647). All the procedures were carried out as humane as possible and were in compliance with relevant ARRIVE guidelines.

### 2.6 Data analysis

All data were expressed as mean ± SEM of *n* number of animals in isometric tension recording experiment. The relaxation responses were expressed as percentage inhibition of the respective contractile agonist‐induced contraction. Maximum response (*E*_max_) and EC_50_ were obtained from non‐linear regression fit curve. pEC_50_ (negative logarithm of EC_50_) values were also determined.

In the calcium imaging experiments, n is the number of DRG cells used. Neurones that exhibited an increase of less than 0.1 from basal ratio after the superfusion with KCl, were treated as non‐viable and excluded from data collection. The mean 340/380 nm ratio represented the [Ca^2+^]_i,_ while the changes in [Ca^2+^]_i_ were expressed as a percentage of the initial KCl response (first addition at 30 mM). Neurones displaying less than 10% of KCl responses were treated as non‐responsive to compounds.

For fluorometric intracellular calcium recordings, *n* is the number of independent experiment performed on cultured cells. The amplitude of [Ca^2+^]_i_ recordings was measured for 50 s, 90–140 s (TRPC3 and TRPC6), 110–130 s (TRPC4) or 150–180 s (TRPC5), respectively. The data was analysed according to height of the plateau as it peaked at different time points for different cells/channels/activators. Data were normalised to the response in the activator control to remove unwanted variation. Concentration‐response curves were fitted with a 3‐parameter nonlinear regression fit. IC_50_ values for TRPC3/4/5/6 channels were obtained from the curve fit.

Statistical analyses were performed on experiments with at least *n* = 5. The study and analysis were carried out randomised, blinded, of equal group sizes unless stated in the respective results. Unpaired Student's *t‐*test was used to compare between vehicle control and treatment groups. In multi‐group analysis, one‐way ANOVA followed by Dunnett's multiple comparisons test was used only when F in ANOVA was significant (*p <* .05). Data were analysed using GraphPad Prism Version 8.2.1 (La Jolla California USA). A probability of less than .05 (*p <* .05) was considered statistically significant, indicated by asterisks: *p < .05; **p < .01; ***p < .001, ****p < .0001.

## 3 RESULTS

### 3.1 Schwarzinicine A exhibits overt vasorelaxation effect

Relaxation magnitude elicited by schwarzinicine A is significantly higher than all the calcium channel blockers tested in this study (see relative *E*_max_ in Table 1). Vehicle control only evoked minimal tissue relaxation (Table 1). Interestingly, Schwarzinicine A has similar pEC_50_ to the TRPC channel inhibitors SKF96365, Pyr3 and HC‐070 (i.e., EC_50_ values differ by less than an order of magnitude; Table 1) but is significantly less potent when compared with nifedipine (Table 1, Figure 1b). A representative trace of aortic relaxation elicited by schwarzinicine A is depicted in Figure 1c.

**TABLE 1:** Vasorelaxation effects of schwarzinicine A and calcium channel blockers

Compounds	*E*_max_ (%)	Relative *E*_max_	pEC_50_
DMSO	39.2 ± 10.3**** (*p <* .0001)	0.3	7.3 ± 0.4 (*p =* .1030)
Schwarzinicine A	123.1 ± 11.7	1	6.1 ± 0.3
SKF96365	76.2 ± 7.2** (*p =* .0033)	0.6	6.7 ± 0.5 (*p =* .7280)
Nifedipine	94.1 ± 6.0 (*p =* .0636)	0.8	7.8 ± 0.2** (*p =* .0060)
Pyr3	67.2 ± 5.3*** (*p =* .0005)	0.5	6.6 ± 0.3 (*p =* .8293)
HC‐070	90.5 ± 1.9* (*p =* .0409)	0.7	6.9 ± 0.5 (*p =* .4426)

*Note*: *E*_max_ of each calcium channel blocker was expressed relative to the corresponding *E*_max_ of schwarzinicine A and presented as the relative *E*_max_. Tissue relaxations were expressed as the percentage of phenylephrine‐induced contraction. The data represent the mean values ± SEM of n number of animals. [One‐way ANOVA followed by Dunnett's multiple comparison test, ****(*p* < 0.0001), ***(*p < *.001), **(*p < *.01) and *(*p < *.05) vs. schwarzinicine A.]

### 3.2 Schwarzinicine A attenuates the contractile response to CaCl_2_ in calcium‐free Krebs solution

**FIGURE 2:**
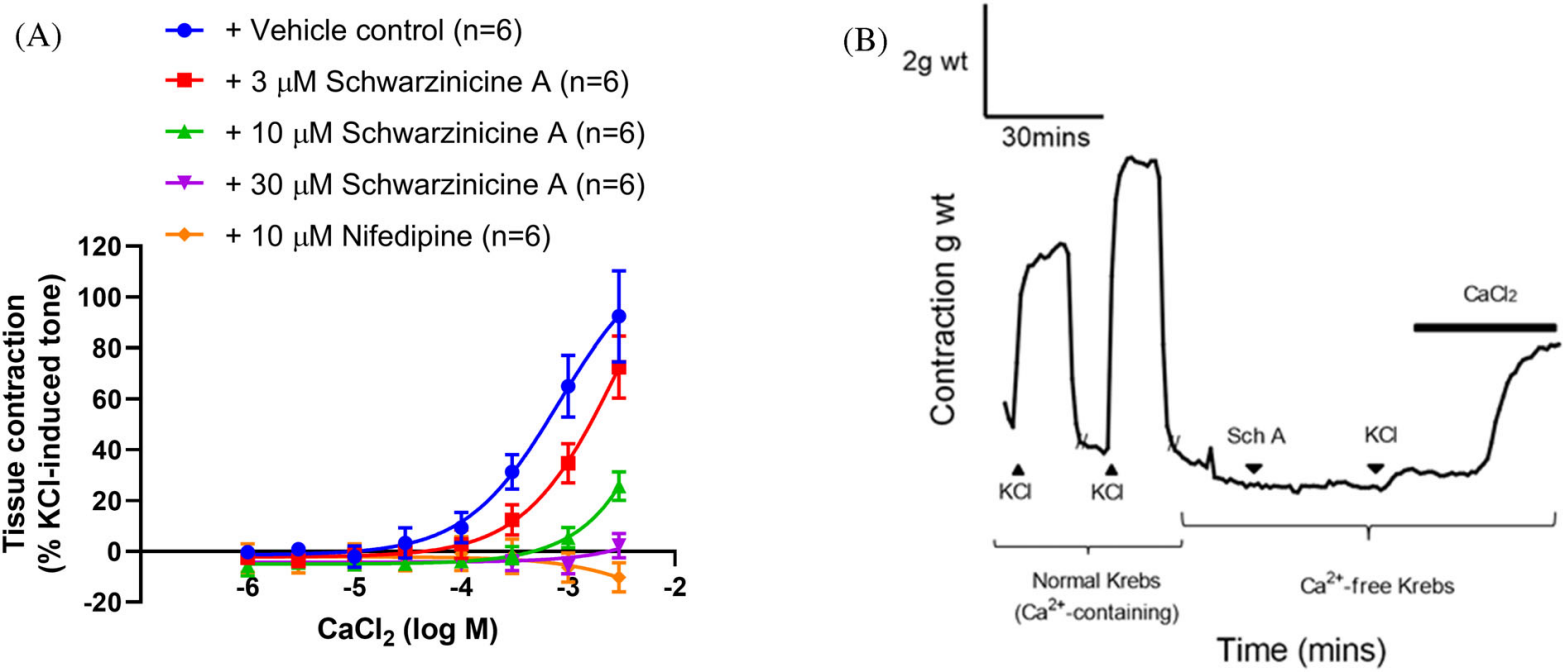
(a) Effects of schwarzinicine A (3, 10 and 30 μM) on CaCl_2_‐induced contraction in calcium‐free Krebs solution. (b) Representative trace recording for calcium re‐addition experiments. The contractile responses were expressed as the percentage of 60 mM KCl‐induced contraction tone in calcium‐containing Krebs solution. The data represent the mean values ± SEM of n number of animals. Significant reductions in CaCl_2_‐induced contractions were shown on those aortic tissues treated with 10 μM and 30 μM of schwarzinicine A and 10 μM nifedipine

Effects of schwarzinicine A on calcium CRC were tested against endothelium‐intact aortic tissues. Aortic tissues treated with vehicle (0.165% DMSO v/v; *E*_max_: 92.4 ± 17.9%) and nifedipine served as the controls. In calcium‐free Krebs solution, nifedipine totally abolished the contractile responses upon re‐addition of CaCl_2_ to the system (*E*_max_: −10.3 ± 5.7%; *p <* .0001 vs. vehicle control; Figure 2). The contractile response to CaCl_2_ following pre‐treatment with schwarzinicine A was concentration‐dependent, with total abolishment observed in the presence of 30 μM schwarzinicine A (*E*_max_ 3 μM: 72.4 ± 12.2%; *p =* .4857; 10 μM: 25.7 ± 5.7%; *p =* .0006; 30 μM: 2.2 ± 4.8%; *p <* .0001; vs. vehicle control). At 10 μM, nifedipine was much better at suppressing the calcium‐induced responses when compared to schwarzinicine A.

### 3.3 Schwarzinicine A attenuates KCl peak response in rat DRG neurones

**FIGURE 3:**
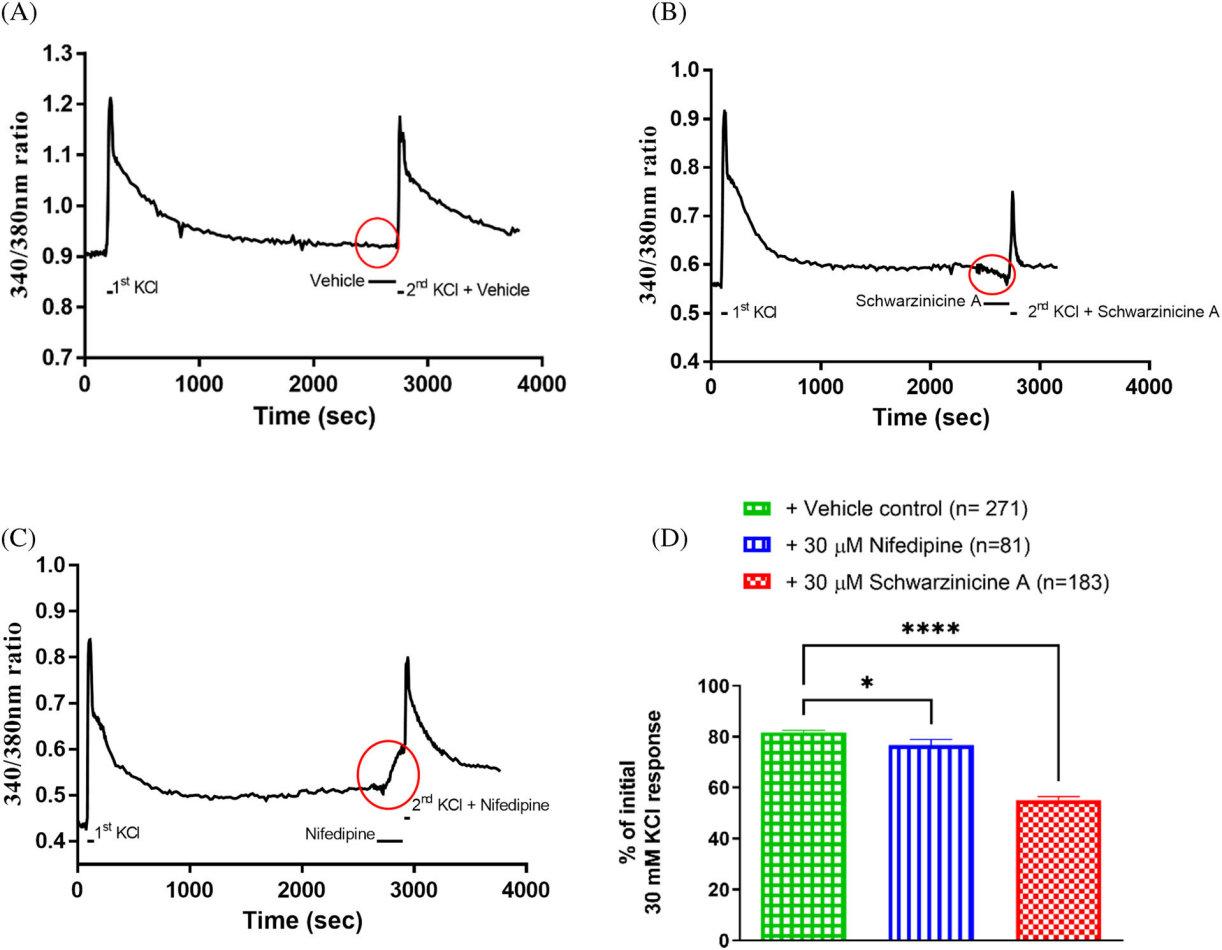
Representative calcium imaging traces of (a) vehicle control‐, (b) schwarzinicine A‐, and (c) nifedipine‐treated neuron cells for the entire experimental period. The black bars below the traces indicate the addition of compounds. (d) Effects of schwarzinicine A and nifedipine on KCl peak response, along with their effects during infusion. All data were expressed as a percentage of the initial KCl response. The data represent the mean values ± SEM of n number of neurons. [One‐way ANOVA followed by Dunnett's multiple comparison test, ****(*p <* .0001) and *(*p <* .05) vs. vehicle control]

Following the observation that schwarzinicine A attenuated the calcium‐induced contractions in the rat aorta, calcium imaging experiments in DRG neurones were carried out to verify the role of schwarzinicine A in modulating calcium dynamics. In intracellular calcium imaging experiments, high potassium (30 mM KCl) produced a sharp peak in the 340/380 nm ratio with an average increment of 0.4 from basal level. Washing with superfusion buffer reversed this response from a depolarising state to basal level. The response of the second KCl addition after incubation with vehicle DMSO, serving as a control, was comparable to the initial KCl response (81.8 ± 0.8%, Figure 3a). Circles in red in Figure 3b,c illustrate the effects of a four‐minute infusion of schwarzinicine A and nifedipine on DRG neuron cells respectively. No influence of vehicle (0.033% DMSO v/v) was observed (Figure 3a,d).

During the four‐minute infusion of schwarzinicine A (30 μM), a slight decrease of 340/380 nm ratio was observed (refer to the circle in red in Figure 3b). Due to the preceding combination with schwarzinicine A, the second KCl peak response was significantly reduced to 55.2 ± 1.3% compared to that of the control KCl response (*p <* .0001, *n* = 183, Figure 3b,d).

The presence of L‐type calcium channels upon depolarisation with high KCl was investigated using nifedipine. Nifedipine marginally attenuated the KCl peak response to 76.7 ± 2.3% compared to control (*p <* .05, Figure 3c,d). During the infusion with nifedipine, a gradual and slow basal calcium elevation, indicated by the 340/380 nm ratio, was observed (refer to the circle in red in Figure 3c). Interestingly, this observation was in contrast to what schwarzinicine A exhibited where the latter suppressed calcium movement into the cells. Subsequently, a further sharp peak increase was followed with the addition of combined KCl and nifedipine (Figure 3c). In this assay, schwarzinicine A exhibited a larger effect in suppressing calcium dynamics compared to nifedipine in the neuronal cells.

### 3.4 Schwarzinicine A inhibits TRPC3, TRPC4, TRPC5 and TRPC6 channels

**FIGURE 4:**
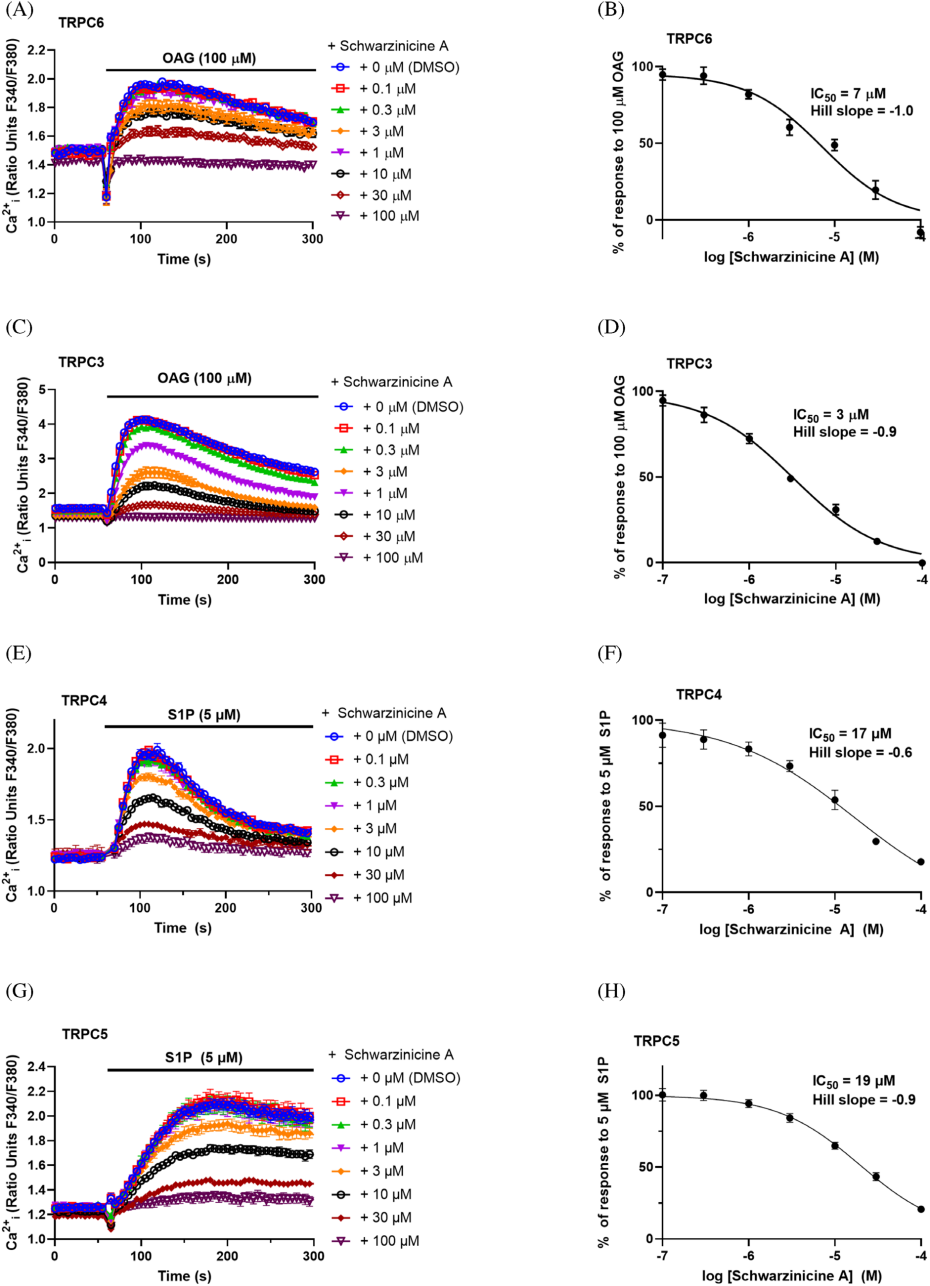
Schwarzinicine A inhibits calcium influx via TRPC channels. Representative [Ca^2+^]_i_ measurements recorded in a single 96‐well plate (N = 6), measuring inhibition of OAG‐mediated calcium influx (a, c) or S1P‐mediated calcium influx (e, g) by 0.1to 100 μM schwarzinicine A in (a) WT HEK 293 cells over‐expressing hTRPC6, (c) (Tet+) HEK T‐REx™ cells over‐expressing hTRPC3, (e) HEK T‐RExTM cells overexpressing hTRPC4 and (g) HEK T‐RExTM overexpressing hTRPC5. Concentration‐response curves to schwarzinicine A were plotted for experiments in (b) hTRPC6, (d) hTRPC3, (f) hTRPC4 and (h) hTRPC5. Responses were calculated at 90–140 s (a–d; hTRPC3/6), 110–130 s (e/f; hTRPC4) or 150–180 s (g/h; hTRPC5) compared to baseline [Ca^2+^]_i_ at 0–55 s. Data are shown as mean ± SEM (hTRPC6: n/N = 5/30; hTPRC3: n/N = 5/30, hTPRC4: n/N = 5/30, hTPRC5: n/N = 5/30)

WT HEK 293 cells transfected with hTRPC6 were used to investigate the effects of schwarzinicine A on TRPC6 channels. Prior to the experiment, cells were loaded with calcium‐selective fluorescent sensor, fura‐2 a.m., to detect the changes in [Ca^2+^]_i_. The cells were pre‐incubated with vehicle control or schwarzinicine A (0.1–100 μM) 30 min before the experiment, and the concentration of vehicle or schwarzinicine A was maintained throughout the experiment. At 60 s, application of 100 μM OAG caused an increase in [Ca^2+^]_i_ due to hTRPC6 activation, and this response was reduced by pre‐incubation with schwarzinicine A in a concentration‐dependent manner (Figure 4a). At the highest concentration tested (100 μM), schwarzinicine A completely inhibited the influx of calcium through hTRPC6 in response to OAG. Our data reveal that the concentration required for half‐maximal inhibition (IC_50_) was 7 μM (pIC_50_ 5.1), with Hill slope = −1.0 (Figure 4b).

Similar protocols were followed to investigate the activity of schwarzinicine A against hTRPC3‐overexpressing (Tet+) HEK 293 T‐REx™ cells. hTRPC3‐mediated calcium entry was evidently reduced by schwarzinicine A (Figure 4c). Complete inhibition was observed at 100 μM. The IC_50_ for TRPC3 was 3 μM (pIC_50_ 5.5), with Hill slope = −0.9 (Figure 4d). These data suggest that schwarzinicine A is able to inhibit both homomeric TRPC3:C3 and TRPC6:C6 channels, with similar potency.

Effects of schwarzinicine A on TRPC4 and TRPC5 channel activity were tested on hTRPC4 or hTRPC5‐overexpressing (Tet+) HEK T‐REx™ cells, using similar protocols to those described for hTRPC3 and hTRPC6. Potent and specific small‐molecule activators of TRPC4 and 5 channels are available (e.g., the natural product (−)‐englerin A) (Akbulut et al., 2015). However, in order to compare effects on vascular relaxation and TRPC channel modulation by schwarzinicine A, we favoured the use of the more physiologically‐relevant TRPC4/5 activator S1P in these assays. S1P is an endogenous signalling molecule that has been shown to activate TRPC5 channels in human VSMCs (Xu et al., 2006). As S1P does not give calcium responses in HEK cells lacking TRPC4/5 expression (Xu et al., 2006), it is suitable for the profiling of potential TRPC4/5 inhibitors.

S1P‐evoked calcium influx mediated by hTRPC4 and hTRPC5 were inhibited by schwarzinicine A (Figure 4e,g) in a concentration‐dependent manner, with half‐maximal inhibition at 17 μM (hTRPC4) (Figure 4f) and 19 μM (hTRPC5) (Figure 4h). Unlike hTRPC3 and hTRPC6, 100 μM schwarzinicine A (the highest concentration tested) did not fully inhibit responses to S1P. These data show that as well as inhibiting TRPC3/6 channels, schwarzinicine A can also inhibit both TRPC4 and TRPC5 channels.

## 4 DISCUSSIONS

In an earlier report from this laboratory (Krishnan et al., 2020), we have shown the overt vasorelaxation effect of schwarzinicine A. This follow‐up study was designed to determine the mechanism of action of its vasorelaxant effect with a specific focus on the mobilisation of intracellular calcium in VSMC. In some earlier reports, activation of TRPC channels has been proposed to induce endothelium‐dependent vasorelaxation via activation of endothelial nitric oxide synthase (eNOS), production of nitric oxide (NO) or downstream hyperpolarisation through calcium‐activated potassium (K_Ca_) channels (Earley & Brayden, 2015; Kochukov et al., 2012; Kwan et al., 2009). However, our findings excluded these vasorelaxant mechanisms of schwarzinicine A, including its involvement in the regulation of endothelium, NO, nucleotide second messengers and potassium channels. The plausible effects of schwarzinicine A affecting the adrenoceptors have also been ruled out (see Supporting Information).

Schwarzinicine A exhibited the highest efficacy as a vasorelaxant amongst all compounds tested. It is interesting to note that its relaxation magnitude is significantly higher than that of nifedipine but with a lower potency recorded. In VSMC, L‐type VGCC is widely known as the major calcium‐permeable channel in the regulation of smooth muscle contraction. In addition, the expression of TRPC channels in VSMC and its role in modulating intracellular calcium hemostasis has been recognised (Bacsa, Tiapko, Stockner, & Groschner, 2020; Mederos y Schnitzler, Gudermann, & Storch, 2018). Schwarzinicine A reversed phenylephrine‐induced tissue contraction, suggesting that it may inhibit downstream L‐type VGCC and TRPC channels as these channels are involved in the vasocontraction induced by phenylephrine (Fransen et al., 2015).

When comparing the vasorelaxation profile of schwarzinicine A with SKF96365, Pyr3 and HC‐070 (Just et al., 2018), all compounds share similar potency (pEC_50_ values) in the micromolar range. The rank of vasorelaxant response (*E*_max_) for these compounds is: schwarzinicine A > HC‐070 > SKF96365 > Pyr3. Pyr3 has been reported as a selective TRPC3 channel inhibitor (IC_50_ 0.8 μM). HC‐070 is a selective, nanomolar inhibitor of TRPC4/5 channels (IC_50_ values of 46 nM for TRPC4 and 9.3 nM for TRPC5), but also inhibits TRPC3 channels at micromolar concentrations (IC_50_ 1.0 uM) (Just et al., 2018; Kiyonaka et al., 2009). SKF96365 has been characterised as a non‐selective TRPC channel inhibitor (IC_50_: 5–25 μM) (Inoue et al., 2001; Merritt et al., 1990; Okada et al., 1998). In our experiments, the EC_50_ value obtained for Pyr3 was similar to other studies, suggesting that Pyr3‐induced relaxation in the rat aorta was mediated by blocking the TRPC3 channel expressed on the smooth muscle of the vasculature. On the other hand, the EC_50_ obtained for HC‐070 in the organ bath experiment is similar to its reported IC_50_ for TRPC3/4/5 channels (Just et al., 2018). This suggests that the vasorelaxation effect of HC‐070 is through inhibition of all these three channels. The role of TRPC3 channel appears to be dominant in vascular relaxation, compared to the role of TRPC4 and 5 channels, as no relaxation effect was observed at nanomolar concentrations of HC‐070. However, we postulate that inhibition of TRPC3 alone could produce a partial but apparent vasorelaxation response. Non‐selective inhibition of multiple TRPC channels as elicited by SKF96365 may be required to observe a maximal vascular response. Schwarzinicine A appears to be inhibiting multiple TRPC channels and L‐type VGCCs, hence giving a large magnitude of relaxation compared to TRPC‐selective compounds and L‐type VGCC blockers.

From the above results, the focus was then moved to verify the role of schwarzinicine A in regulating calcium mobilisation in rat aorta. Depolarisation of the plasma membrane following depletion of external calcium mimics the condition of the store‐operated calcium entry pathway. In a calcium‐free environment, schwarzinicine A markedly suppressed the contractile response to the re‐addition of CaCl_2_ in a concentration‐dependent manner, affecting calcium entry in a similar way to nifedipine, a potent L‐type VGCC inhibitor. In rat DRG cells, KCl‐stimulated VGCC via membrane depolarisation appears that L‐type VGCC‐insensitive calcium influx seems to be dominant compared to the L‐type VGCC‐sensitive route. This is suggested as the incubation with nifedipine did not suppress KCl‐induced contraction as much as schwarzinicine A. Most importantly, this finding proposed the notion that the inhibitory action of schwarzinicine A in calcium dynamics is not entirely the same as that of nifedipine but could involve a combination of different calcium channels. These observations indicate that schwarzinicine A affected calcium influx via a mechanism different from a pure L‐type calcium channel blocker like nifedipine. However the evidence gathered did not rule out its inhibition on L‐type VGCCs as KCl‐induced depolarisation involved L‐type VGCCs and store‐operated TRP (Lopez et al., 2020; Ratz & Berg, 2006). Schwarzinicine A may block a broad spectrum of Ca^2+^ permeable channels, including but not limited to TRPC channels. In order to measure the intracellular calcium dynamics by schwarzinicine A in TRPC channels, HEK cells overexpressing human TRPC3/4/5/6 channels were employed. Schwarzinicine A inhibits calcium influx through homomeric hTRPC3, 4, 5 and 6 channels at low micromolar range. To date, there are some small molecules identified as TRPC6 inhibitors such as DS88790512 and ribemansides A/B but with no information on their selectivity against other TRPC subtypes (Motoyama et al., 2018; Zhou et al., 2018). TRPC proteins are able to form heterotetrameric channels, particularly among TRPC1/4/5 and TRPC3/6/7, owing to their closely related homologues (Hofmann, Schaefer, Schultz, & Gudermann, 2002). Different TRPC tetramers can have distinct biophysical properties, pharmacology and function (Minard et al., 2018; Wang et al., 2020). Modulation of endogenous TRPC channels is complex and involves complex interplay with endogenous and dietary lipids (Svobodova & Groschner, 2016). Lipid modulation may be indirect; for example, S1P can activate channels (at least in part) through G‐protein signalling (Xu et al., 2006). However, lipids may also act on TRPC channels directly. For example, DAGs have been shown to activate TRPC3/6/7 channels directly, independently of PKC activation (Beck et al., 2006; Hofmann et al., 1999), potentially via a proposed agonist binding site identified on TRPC6 (Bai et al., 2020). Lysophospholipids such as lysophosphatidylcholine (LPC) can modulate TRPC5 channel directly, potentially through direct interaction with the phospholipid bilayer or at lipid interaction sites of these channels (Flemming et al., 2006; Wright et al., 2020). From our results, the higher potency of schwarzinicine A on TRPC3 and TRPC6 inhibition could be due to the greater involvement they have in the regulation of vasoconstriction and myogenic responses compared to TRPC4 and TRPC5 (Álvarez‐Miguel et al., 2017; Martín‐Bórnez et al., 2020).

Homotetramers of TRPC3 or TRPC6 are mostly formed in heterologous overexpression HEK 293 cells, however formation of heteromultimeric channels in vivo with different association patterns may alter their functional properties (Hofmann et al., 2002). TRPC3 was found to be upregulated in TRPC6‐knockout mice when unexpected enhancement in vascular reactivity was detected (Dietrich et al., 2005), suggesting their functional roles may overlap to compensate for missing mechanisms. Despite possessing similar pore properties, they have different affinities towards calcium (Boulay et al., 1997; Zhu et al., 1996) which lead to distinct functional roles in regulating vascular smooth muscle contractility and other systems (Dietrich et al., 2005; Klein et al., 2020). Distinction between functions of TRPC3 and TRPC6 was supported by another study which demonstrated that TRPC3 mediates agonist‐induced depolarisation in cerebral artery SMC, while TRPC6 plays a role in pressure‐induced depolarisation, which potentiates myogenic tone (Reading, Earley, Waldron, Welsh, & Brayden, 2005). Therefore, the effect of schwarzinicine A on heteromeric TRPCs warrants further studies. Future patch‐clamp electrophysiology experiments and/or photoaffinity labelling would also be useful to provide further evidence on the link between schwarzinicine A and TRPC channel inhibition in smooth muscle cells (Bauer et al., 2020). Its high temporal resolution allows determination of drug reversibility, voltage‐dependency, and binding site (extracellular or intracellular, or both).

In conclusion, schwarzinicine A as a novel phenylethylamine, has elicited an overt concentration‐dependent relaxation response in the rat aorta. It displayed a calcium inhibitory property by blocking calcium entry in both the vascular function and calcium imaging measurement in rat DRG cells. This study also revealed schwarzinicine A as a non‐selective TRPC channel blocker. Nevertheless, it is possible that schwarzinicine A inhibits more than one family of TRP channels. Its ability to evoke marked vasorelaxation responses in aortic smooth muscle tissues could potentially be clinically relevant in cases of dysregulation of calcium‐dependent vascular smooth muscle that is mediated through L‐type VGCCs and TRPC channels. Finally, schwarzinicine A could be used as a potential candidate to facilitate the development of analogues to investigate the structure–activity relationships of TRPC channels.

## AUTHOR CONTRIBUTIONS

Yin‐Ying Mak, Bi‐Juin Loong, Paul Millns, Fong‐Kai Lee, Claudia C. Bauer performed the experiments. Kang‐Nee Ting, Yvonne Mbaki, Kuan‐Hon Lim, Cin Kong, Claudia C. Bauer designed the research study. Kang‐Nee Ting, Kuan‐Hon Lim, Robin S. Bon, Paul Millns, Yvonne Mbaki contributed essential reagents or tools. Yin‐Ying Mak, Bi‐Juin Loong, Kang‐Nee Ting, Yvonne Mbaki, Cin Kong, Claudia C. Bauer, Robin S. Bon analysed the data. All authors wrote and reviewed the paper.

## CONFLICT OF INTEREST

The authors declare that they have no conflict of interest.

## Supporting information


**Appendix S1** Supporting Information

## Data Availability

The data that support the findings of this study are available on request from the corresponding author. The data are not publicly available due to privacy or ethical restrictions.

## References

[ptr7489-bib-0001] Akbulut, Y. , Gaunt, H. J. , Muraki, K. , Ludlow, M. J. , Amer, M. S. , Bruns, A. , … Waldmann, H. (2015). Englerin a is a potent and selective activator of TRPC4 and TRPC5 calcium channels. Angewandte Chemie ‐ International Edition, 54(12), 3787–3791. 10.1002/anie.201411511 25707820 PMC7116557

[ptr7489-bib-0002] Álvarez‐Miguel, I. , Cidad, P. , Pérez‐García, M. T. , & López‐López, J. R. (2017). Differences in TRPC3 and TRPC6 channels assembly in mesenteric vascular smooth muscle cells in essential hypertension. The Journal of Physiology, 595(5), 1497–1513. 10.1113/JP273327 27861908 PMC5330869

[ptr7489-bib-0003] Bacsa, B. , Tiapko, O. , Stockner, T. , & Groschner, K. (2020). Mechanisms and significance of Ca^2+^ entry through TRPC channels. Current Opinion in Physiology, 17, 25–33. 10.1016/j.cophys.2020.06.005 33210055 PMC7116371

[ptr7489-bib-0004] Bai, Y. , Yu, X. , Chen, H. , Horne, D. , White, R. , Wu, X. , … Huang, X. (2020). Structural basis for pharmacological modulation of the TRPC6 channel. eLife, 9, 1–18. 10.7554/eLife.53311 PMC7082128 32149605

[ptr7489-bib-0005] Bauer, C. C. , Minard, A. , Pickles, I. B. , Simmons, K. J. , Chuntharpursat‐Bon, E. , Burnham, M. P. , … Bon, R. S. (2020). Xanthine‐based photoaffinity probes allow assessment of ligand engagement by TRPC5 channels. RSC Chemical Biology, 1(5), 436–448. 10.1039/D0CB00126K PMC10655067 41324141

[ptr7489-bib-0006] Beck, B. , Zholos, A. , Sydorenko, V. , Roudbaraki, M. , Lehen'kyi, V. , Bordat, P. , … Skryma, R. (2006). TRPC7 is a receptor‐operated DAG‐activated channel in human keratinocytes. Journal of Investigative Dermatology, 126(9), 1982–1993. 10.1038/sj.jid.5700352 16741513

[ptr7489-bib-0007] Boulay, G. , Zhu, X. , Peyton, M. , Jiang, M. , Hurst, R. , Stefani, E. , & Birnbaumer, L. (1997). Cloning and expression of a novel mammalian homolog of drosophila transient receptor potential (Trp) involved in calcium entry secondary to activation of receptors coupled by the G_q_ class of G protein. Journal of Biological Chemistry, 272(47), 29672–29680. 10.1074/jbc.272.47.29672 9368034

[ptr7489-bib-0008] Chen, X. , Yang, D. , Ma, S. , He, H. , Luo, Z. , Feng, X. , … Zhu, Z. (2010). Increased rhythmicity in hypertensive arterial smooth muscle is linked to transient receptor potential canonical channels. Journal of Cellular and Molecular Medicine, 14(10), 2483–2494. 10.1111/j.1582-4934.2009.00890.x 19725917 PMC3823165

[ptr7489-bib-0009] Dietrich, A. , Mederos y Schnitzler, M. , Gollasch, M. , Gross, V. , Storch, U. , Dubrovska, G. , … Birnbaumer, L. (2005). Increased vascular smooth muscle contractility in TRPC6^−/−^ mice. Molecular and Cellular Biology, 25(16), 6980–6989. 10.1128/MCB.25.16.6980-6989.2005 16055711 PMC1190236

[ptr7489-bib-0010] Earley, S. , & Brayden, J. E. (2015). Transient receptor potential channels in the vasculature. Physiological Reviews, 95(1), 645–690. 10.1152/physrev.00026.2014 25834234 PMC4551213

[ptr7489-bib-0011] Facemire, C. S. , Mohler, P. J. , & Arendshorst, W. J. (2004). Expression and relative abundance of short transient receptor potential channels in the rat renal microcirculation. American Journal of Physiology‐Renal Physiology, 286(3), F546–F551. 10.1152/ajprenal.00338.2003 14678949

[ptr7489-bib-0012] Flemming, P. K. , Dedman, A. M. , Xu, S. Z. , Li, J. , Zeng, F. , Naylor, J. , … Beech, D. J. (2006). Sensing of lysophospholipids by TRPC5 calcium channel. Journal of Biological Chemistry, 281(8), 4977–4982. 10.1074/jbc.M510301200 16368680

[ptr7489-bib-0013] Fransen, P. , Van Hove, C. E. , Leloup, A. J. A. , Martinet, W. , De Meyer, G. R. Y. , Lemmens, K. , … Schrijvers, D. M. (2015). Dissecting out the complex Ca^2+^‐mediated phenylephrine‐induced contractions of mouse aortic segments. PLoS One, 10(3), 1–17. 10.1371/journal.pone.0121634 PMC4372603 25803863

[ptr7489-bib-0014] Harteneck, C. , Klose, C. , & Krautwurst, D. (2011). Synthetic modulators of TRP channel activity. In Advances in experimental medicine and biology (Vol. 704, pp. 87–106). Dordrecht: Springer. 10.1007/978-94-007-0265-3_4 21290290

[ptr7489-bib-0015] Hofmann, T. , Obukhov, A. G. , Schaefer, M. , Harteneck, C. , Gudermann, T. , & Schultz, G. (1999). Direct activation of human TRPC6 and TRPC3 channels by diacylglycerol. Nature, 397(6716), 259–263. 10.1038/16711 9930701

[ptr7489-bib-0016] Hofmann, T. , Schaefer, M. , Schultz, G. , & Gudermann, T. (2002). Subunit composition of mammalian transient receptor potential channels in living cells. Proceedings of the National Academy of Sciences of the United States of America, 99(11), 7461–7466. 10.1073/pnas.102596199 12032305 PMC124253

[ptr7489-bib-0017] Inoue, R. , Okada, T. , Onoue, H. , Hara, Y. , Shimizu, S. , Naitoh, S. , … Mori, Y. (2001). The transient receptor potential protein homologue TRP6 is the essential component of vascular alpha_1_‐adrenoceptor‐activated Ca^2+^‐permeable cation channel. Circulation Research, 88(3), 325–332. 10.1161/01.RES.88.3.325 11179201

[ptr7489-bib-0018] Jung, S. , Strotmann, R. , Schultz, G. , & Plant, T. D. (2002). TRCP6 is a candidate channel involved in receptor stimulated cation currents in A7r5 smooth muscle cells. American Journal of Physiology ‐ Cell Physiology, 282(2), C347–C359. 10.1152/ajpcell.00283.2001 11788346

[ptr7489-bib-0019] Just, S. , Chenard, B. L. , Ceci, A. , Strassmaier, T. , Chong, J. A. , Blair, N. T. , … Moran, M. M. (2018). Treatment with HC‐070, a potent inhibitor of TRPC4 and TRPC5, leads to anxiolytic and antidepressant effects in mice. PLoS One, 13(1), e0191225. 10.1371/journal.pone.0191225 29385160 PMC5791972

[ptr7489-bib-0020] Kiselyov, K. , Xu, X. , Mozhayeva, G. , Kuo, T. , Pessah, I. , Mignery, G. , … Muallem, S. (1998). Functional interaction between InsP_3_ receptors and store‐operated Htrp3 channels. Nature, 396(6710), 478–482. 10.1038/24890 9853757

[ptr7489-bib-0021] Kiyonaka, S. , Kato, K. , Nishida, M. , Mio, K. , Numaga, T. , Sawaguchi, Y. , … Mori, Y. (2009). Selective and direct inhibition of TRPC3 channels underlies biological activities of a pyrazole compound. Proceedings of the National Academy of Sciences of the United States of America, 106(13), 5400–5405. 10.1073/pnas.0808793106 19289841 PMC2664023

[ptr7489-bib-0022] Klein, S. , Mentrup, B. , Timmen, M. , Sherwood, J. , Lindemann, O. , Fobker, M. , … Stange, R. (2020). Modulation of transient receptor potential channels 3 and 6 regulates osteoclast function with impact on trabecular bone loss. Calcified Tissue International, 106(6), 655–664. 10.1007/s00223-020-00673-8 32140760

[ptr7489-bib-0023] Kochukov, M. Y. , Balasubramanian, A. , Noel, R. C. , & Marrelli, S. P. (2012). Role of TRPC1 and TRPC3 channels in contraction and relaxation of mouse thoracic aorta. Journal of Vascular Research, 50(1), 11–20. 10.1159/000342461 23095462 PMC3556789

[ptr7489-bib-0024] Krishnan, P. , Lee, F. K. , Yap, V. A. , Low, Y. Y. , Kam, T. S. , Yong, K. T. , … Lim, K. H. (2020). Schwarzinicines A‐G, 1,4‐diarylbutanoid‐phenethylamine conjugates from the leaves of *Ficus schwarzii* . Journal of Natural Products, 83(1), 152–158. 10.1021/acs.jnatprod.9b01160 31935094

[ptr7489-bib-0025] Kunichika, N. , Yu, Y. , Remillard, C. V. , Platoshyn, O. , Zhang, S. , & Yuan, J. X. J. (2004). Overexpression of TRPC1 enhances pulmonary vasoconstriction induced by capacitative Ca^2+^ entry. American Journal of Physiology ‐ Lung Cellular and Molecular Physiology, 287(5 31–5), 962–969. 10.1152/ajplung.00452.2003 15220115

[ptr7489-bib-0026] Kwan, H. Y. , Shen, B. , Ma, X. , Kwok, Y. C. , Huang, Y. , Man, Y. B. , … Yao, X. (2009). TRPC1 associates with BKCa channel to form a signal complex in vascular smooth muscle cells. Circulation Research, 104(5), 670–678. 10.1161/CIRCRESAHA.108.188748 19168436

[ptr7489-bib-0027] Lee, F. K. , Krishnan, P. , Muhamad, A. , Low, Y.‐Y. , Kam, T.‐S. , Ting, K.‐N. , & Lim, K.‐H. (2021). Concise synthesis of the vasorelaxant alkaloids schwarzinicines A and B. Natural Product Research. 10.1080/14786419.2021.1903005 33749454

[ptr7489-bib-0028] Lee, M.‐K. , Millns, P. , Mbaki, Y. , Ng, S.‐T. , Tan, C.‐S. , Lim, K.‐H. , … Ting, K.‐N. (2018). Data on the Lignosus rhinocerotis water soluble sclerotial extract affecting intracellular calcium level in rat dorsal root ganglion cells. Data in Brief, 18, 1322–1326. 10.1016/j.dib.2018.04.033 29900310 PMC5997891

[ptr7489-bib-0029] Liu, D. , Xiong, S. , & Zhu, Z. (2014). Imbalance and dysfunction of transient receptor potential channels contribute to the pathogenesis of hypertension. Science China Life Sciences, 57(8), 818–825. 10.1007/s11427-014-4713-3 25104455

[ptr7489-bib-0030] Loong, B. J. , Tan, J. H. , Lim, K. H. , Mbaki, Y. , & Ting, K. N. (2015). Contractile function of smooth muscle retained after overnight storage. Naunyn‐Schmiedeberg's Archives of Pharmacology, 388(10), 1061–1067. 10.1007/s00210-015-1140-3 26051407

[ptr7489-bib-0031] Lopez, J. J. , Jardin, I. , Sanchez‐Collado, J. , Salido, G. M. , Smani, T. , & Rosado, J. A. (2020). TRPC channels in the SOCE scenario. Cell, 9(1), 126. 10.3390/cells9010126 PMC7016597 31948094

[ptr7489-bib-0032] Martín‐Bórnez, M. , Galeano‐Otero, I. , Del Toro, R. , & Smani, T. (2020). TRPC and TRPV channels' role in vascular remodeling and disease. International Journal of Molecular Sciences, 21(17), 1–17. 10.3390/ijms21176125 PMC7503586 32854408

[ptr7489-bib-0033] Mederos y Schnitzler, M. , Gudermann, T. , & Storch, U. (2018). Emerging roles of diacylglycerol‐sensitive TRPC4/5 channels. Cell, 7(11), 218. 10.3390/cells7110218 PMC6262340 30463370

[ptr7489-bib-0034] Merritt, J. E. , Armstrong, W. P. , Benham, C. D. , Hallam, T. J. , Jacob, R. , Jaxa‐Chamiec, A. , … Rink, T. J. (1990). SK&F 96365, a novel inhibitor of receptor‐mediated calcium entry. Biochemical Journal, 271(2), 515–522. 10.1042/bj2710515 2173565 PMC1149585

[ptr7489-bib-0035] Minard, A. , Bauer, C. , Wright, D. , Rubaiy, H. , Muraki, K. , Beech, D. , & Bon, R. (2018). Remarkable progress with small‐molecule modulation of TRPC1/4/5 channels: Implications for understanding the channels in health and disease. Cell, 7(6), 52. 10.3390/cells7060052 PMC6025525 29865154

[ptr7489-bib-0036] Minard, A. , Bauer, C. C. , Chuntharpursat‐Bon, E. , Pickles, I. B. , Wright, D. J. , Ludlow, M. J. , … Bon, R. S. (2019). Potent, selective, and subunit‐dependent activation of TRPC5 channels by a xanthine derivative. British Journal of Pharmacology, 176(20), 3924–3938. 10.1111/bph.14791 31277085 PMC6811774

[ptr7489-bib-0037] Motoyama, K. , Nagata, T. , Kobayashi, J. , Nakamura, A. , Miyoshi, N. , Kazui, M. , … Sakakura, T. (2018). Discovery of a bicyclo[4.3.0]nonane derivative DS88790512 as a potent, selective, and orally bioavailable blocker of transient receptor potential canonical 6 (TRPC6). Bioorganic & Medicinal Chemistry Letters, 28(12), 2222–2227. 10.1016/j.bmcl.2018.03.056 29752182

[ptr7489-bib-0038] Okada, T. , Shimizu, S. , Wakamori, M. , Maeda, A. , Kurosaki, T. , Takada, N. , … Mori, Y. (1998). Molecular cloning and functional characterization of a novel receptor‐ activated TRP Ca^2+^ channel from mouse brain. Journal of Biological Chemistry, 273(17), 10279–10287. 10.1074/jbc.273.17.10279 9553080

[ptr7489-bib-0039] Ratz, P. H. , & Berg, K. M. (2006). 2‐Aminoethoxydiphenyl borate inhibits KCl‐induced vascular smooth muscle contraction. European Journal of Pharmacology, 541(3), 177–183. 10.1016/j.ejphar.2006.05.014 16765942

[ptr7489-bib-0040] Reading, S. A. , Earley, S. , Waldron, B. J. , Welsh, D. G. , & Brayden, J. E. (2005). TRPC3 mediates pyrimidine receptor‐induced depolarization of cerebral arteries. American Journal of Physiology ‐ Heart and Circulatory Physiology, 288(5, 57–55. 10.1152/ajpheart.00861.2004 15604128

[ptr7489-bib-0041] Svobodova, B. , & Groschner, K. (2016). Mechanisms of lipid regulation and lipid gating in TRPC channels. Cell Calcium, 59(6), 271–279. 10.1016/j.ceca.2016.03.012 27125985

[ptr7489-bib-0042] Wang, H. , Cheng, X. , Tian, J. , Xiao, Y. , Tian, T. , Xu, F. , … Zhu, M. X. (2020). TRPC channels: Structure, function, regulation and recent advances in small molecular probes. Pharmacology & Therapeutics, 209, 107497. 10.1016/j.pharmthera.2020.107497 32004513 PMC7183440

[ptr7489-bib-0043] Welsh, D. G. , Morielli, A. D. , Nelson, M. T. , & Brayden, J. E. (2002). Transient receptor potential channels regulate myogenic tone of resistance arteries. Circulation Research, 90(3), 248–250. 10.1161/hh0302.105662 11861411

[ptr7489-bib-0044] Wright, D. J. , Simmons, K. J. , Johnson, R. M. , Beech, D. J. , Muench, S. P. , & Bon, R. S. (2020). Human TRPC5 structures reveal interaction of a xanthine‐based TRPC1/4/5 inhibitor with a conserved lipid binding site. Communications Biology, 3(1), 704. 10.1038/s42003-020-01437-8 33230284 PMC7683545

[ptr7489-bib-0045] Xu, S. Z. , Muraki, K. , Zeng, F. , Li, J. , Sukumar, P. , Shah, S. , … Beech, D. J. (2006). A sphingosine‐1‐phosphate‐activated calcium channel controlling vascular smooth muscle cell motility. Circulation Research, 98(11), 1381–1389. 10.1161/01.RES.0000225284.36490.a2 16675717 PMC2648505

[ptr7489-bib-0046] Yip, H. , Chan, W. Y. , Leung, P. C. , Kwan, H. Y. , Liu, C. , Huang, Y. , … Yao, X. (2004). Expression of TRPC homologs in endothelial cells and smooth muscle layers of human arteries. Histochemistry and Cell Biology, 122(6), 553–561. 10.1007/s00418-004-0720-y 15538613

[ptr7489-bib-0047] Zhou, B. , Wang, Y. , Zhang, C. , Yang, G. , Zhang, F. , Yu, B. , … Cao, Z. (2018). Ribemansides A and B, TRPC6 inhibitors from *Ribes manshuricum* that suppress TGF‐β1‐induced fibrogenesis in HK‐2 cells. Journal of Natural Products, 81(4), 913–917. 10.1021/acs.jnatprod.7b01037 29469570

[ptr7489-bib-0048] Zhu, X. , Jiang, M. , Peyton, M. , Boulay, G. , Hurst, R. , Stefani, E. , & Birnbaumer, L. (1996). *trp*, a novel mammalian gene family essential for agonist‐activated capacitative Ca^2+^ entry. Cell, 85(5), 661–671. 10.1016/S0092-8674(00)81233-7 8646775

