# Supplementary material for: Schwarzinicine A inhibits transient receptor potential canonical channels and exhibits overt vasorelaxation effects
Source: Phytother Res. 2022 May 10;36(7):2952–63. doi: 10.1002/ptr.7489 (PMC9544403; doi:10.1002/ptr.7489)
Supplement: Supplementary file 1 — Appendix S1 Supporting Information [file PTR-36-2952-s001.docx]

**Supplementary materials**

1. Schwarzinicine A-induced relaxation is NO and endothelium-independent


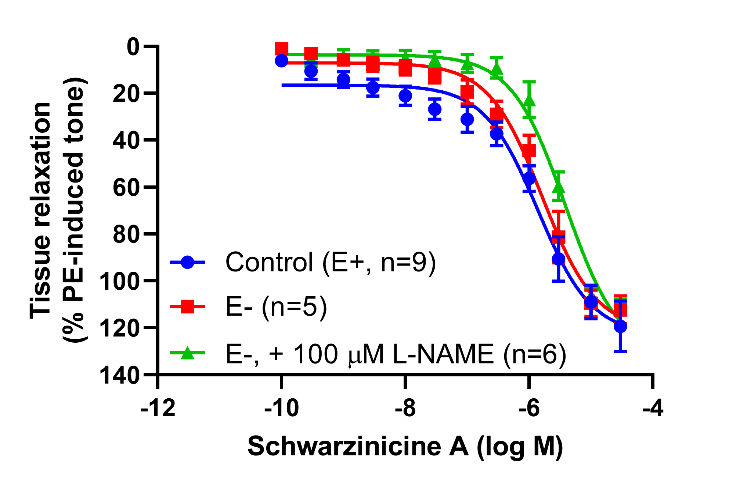


**Figure S1** Schwarzinicine A-induced relaxation is endothelium- and NO-independent. Removal of endothelium (E-) did not affect the relaxation to schwarzinicine A (E_max_: E+, 123.1 ± 11.7%; E-, 123.8 ± 9.5%; *p =* 0.9987; vs control; pEC_50_: E+, 6.1 ± 0.3; E-, 5.8 ± 0.2; *p =* 0.6070; vs control). Presence of L-NAME (100 µM) also did not affect the relaxation profile (E_max_: 132.2 ± 4.6%, *p =* 0.7614; pEC_50_: 5.4 ± 0.1, *p =* 0.0733; vs control). Tissue relaxations were expressed as the percentage of phenylephrine-induced contraction. The data represent the mean values ± SEM of n number of animals. [One-way ANOVA followed by Dunnett’s multiple comparison test showed no significance when comparing the relaxations to schwarzinicine A after endothelium removal and in combined with L-NAME treatment with the control]

1. Schwarzinicine A does not affect adrenoceptors regulationa

**
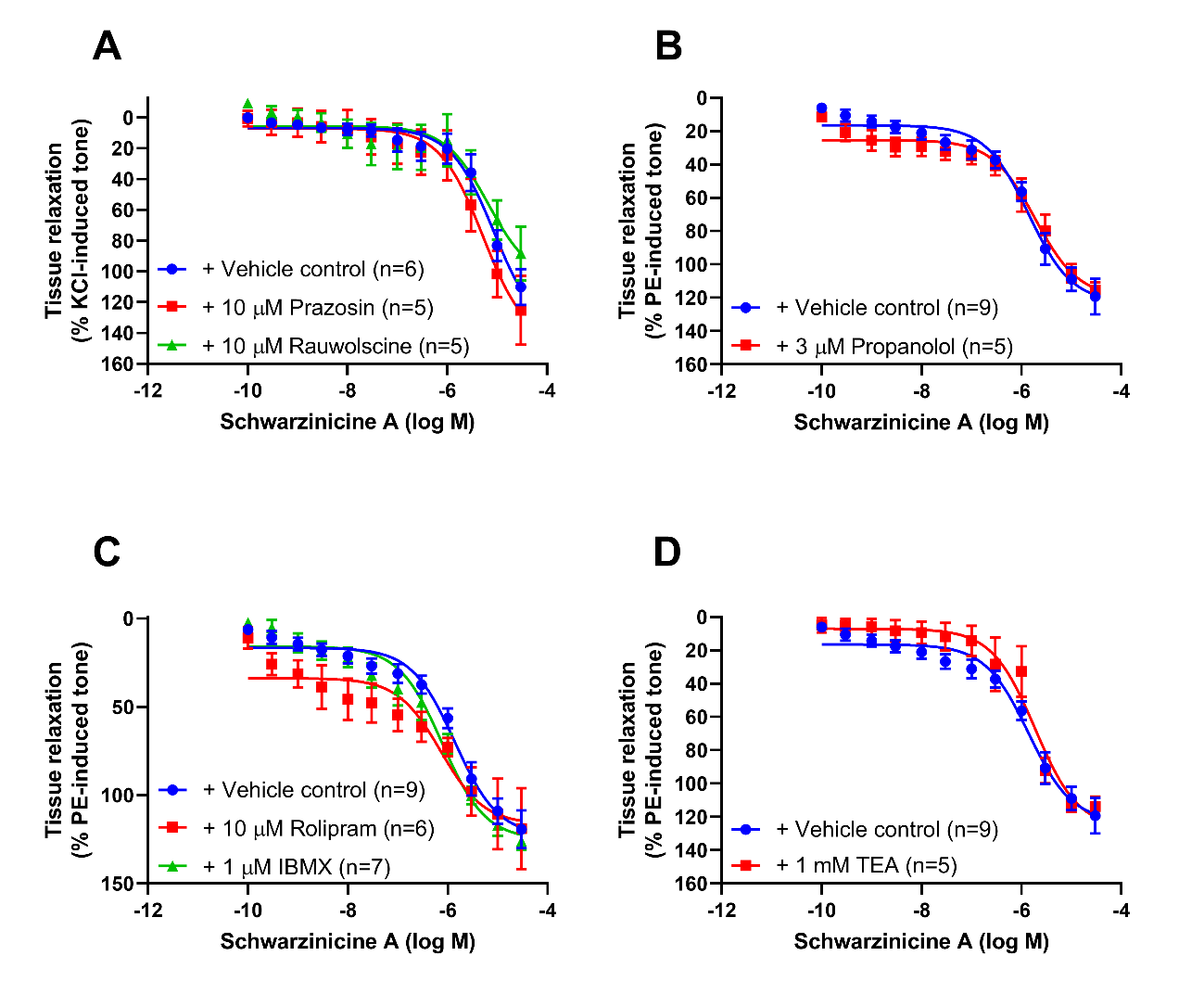
Figure S2** Schwarzinicine A-induced relaxation does not involve adrenoceptors, cyclic nucleotides and potassium channels. Effects of (A) alpha-adrenergic antagonists (prazosin and rauwolscine, 10 µM) and (B) beta-adrenergic antagonists (propranolol, 3 µM), (C) phosphodiesterase inhibitors, (rolipram, 10 µM and IBMX, 1 µM), (D) potassium channel blocker (TEA, 1 mM) on schwarzinicine A-induced relaxation. The relaxations were expressed as the percentage of respective KCl- and phenylephrine-induced contraction.

**Table S1** Summary of the E_max_ and pEC_50_ of schwarzinicine A-induced relaxation when pre-treated with alpha-adrenergic antagonists (prazosin and rauwolscine, 10 µM), beta-adrenergic antagonists (propranolol, 3 µM), phosphodiesterase inhibitors, (rolipram, 10 µM and IBMX, 1 µM), and potassium channel blocker (TEA, 1 mM).

| Pre-incubation compounds | E_max_ (%) | pEC_50_ |
| --- | --- | --- |
| Vehicle control | 110.0 ± 11.5 | 5.6 ± 0.3 |
| Prazosin | 125.2 ± 22.2 (*p =* 0.5742) | 5.7 ± 0.1 (*p =* 0.9611) |
| Rauwolscine | 88.23 ± 17.2 (*p =* 0.7544) | 6.1 ± 0.4 (*p =* 0.3567) |
| Vehicle control | 123.1 ± 11.6 | 6.1 ± 0.3 |
| Propranolol | 126.2 ± 5.7 (*p =* 0.8540) | 5.6 ± 0.2 (*p =* 0.2590) |
| Vehicle control | 123.1 ± 11.7 | 6.1 ± 0.3 |
| Rolipram | 119.5 ± 26.6 (*p =* 0.9805) | 6.8 ± 0.5 (*p =* 0.2790) |
| IBMX | 125.1 ± 6.3 (*p =* 0.9941) | 6.3 ± 0.2 (*p =* 0.9500) |
| Vehicle control | 123.1 ± 11.6 | 6.1 ± 0.3 |
| TEA | 129.3 ± 7.1 (*p =* 0.7175) | 5.8 ± 0.2 (*p =* 0.4266) |

Tissue relaxations were expressed as the percentage of KCl- or phenylephrine-induced contraction. The data represent the mean values ± SEM of n number of animals. [One-way ANOVA followed by Dunnett’s multiple comparison test showed no significance between vehicle control and those with prior treatments with adrenergic antagonists or phosphodiesterase inhibitors. Student’s unpaired t-test showed no significant difference in between vehicle control and propranolol-treated or TEA-treated aortic tissues
